# Magnetic yolk-shell structured periodic mesoporous organosilica supported palladium as a powerful and highly recoverable nanocatalyst for the reduction of nitrobenzenes

**DOI:** 10.1038/s41598-024-66883-4

**Published:** 2024-07-15

**Authors:** Meysam Norouzi, Dawood Elhamifar, Shiva Kargar

**Affiliations:** Department of Chemistry, Yasouj University, Yasouj, 75918-74831 Iran

**Keywords:** Magnetic yolk–shell structured nanocomposite, Periodic mesoporous organosilica, Reduction of nitroarenes, Green conditions, Nanoscale materials, Catalysis

## Abstract

A novel palladium-loaded yolk-shell structured nanomaterial with magnetite core and phenylene-based periodic mesoporous organosilica (PMO) shell (Fe_3_O_4_@YS-Ph-PMO/Pd) nanocatalyst was synthesized for the reduction of nitrobenzenes. The Fe_3_O_4_@YS-Ph-PMO/Pd was prepared through cetyltrimethylammonium bromide (CTAB) directed condensation of 1,4-bis(triethoxysilyl)benzene (BTEB) around Fe_3_O_4_@silica nanoparticles followed by treatment with palladium acetate. This nanocatalyst was characterized by using Fourier transform infrared (FT-IR) spectroscopy, thermal gravimetric analysis (TGA), low-angle and wide-angle powder X-ray diffraction (PXRD), scanning electron microscopy (SEM), transmission electron microscopy (TEM) and vibrating sample magnetometer (VSM) analyses. These analyses showed a magnetic nanomaterial with high chemical and thermal stability for the designed composite. The Fe_3_O_4_@YS-Ph-PMO/Pd nanocomposite was employed as a powerful and highly recoverable catalyst in the green reduction of nitroarenes in H_2_O at room temperature. A variety of nitroarene derivatives were applied as substrate in the presence of 0.9 mol% of Fe_3_O_4_@YS-Ph-PMO/Pd catalyst. All nitroarenes were selectively converted to their corresponding amines with high to excellent yields (92–96%) within short reaction times (10–18 min). This catalyst was recovered and reused at least 11 times without significant decrease in efficiency and stability.

## Introduction

In the recent years, the yolk-shell (YS) structured nanomaterials have demonstrated a new type of structures containing a void between core and shell. These materials have received increasing attention owing to tunable their physiochemical properties and also high capability in the adsorption and catalytic processes^1–9^. These nanomaterials have a lot of application in areas of data storage, catalysis and environmental remediation^5,6,10–12^. Due to the aforementioned notes and also widespread applications of YSs in green chemistry, various methods such as Kirkendall, etching, ship in bottle and Ostwald have been employed for the synthesis of these materials^13–16^. Among these, selective etching is very interested in between researchers^17,18^. YSs with magnetite core and PMO shell (Mag@YS-PMOs) have attracted much research attention because of their combined properties of YSs, magnetic materials and ordered mesoporous structures^12,19–22^. The Mag@YS-PMOs have the advantages of magnetic nanoparticles such as easy magnetic separation, high dispersion ability in aqueous media as well as high chemical reactivity and stability. In addition, these have also the advantages of PMOs such as highly-ordered mesostructure, excellent loading of uniformly distributed organic functions in their framework and high moisture stability^12,19,23^. These characteristics make Mag@YS-PMOs as promising applicant for supporting metals in different chemical processes^5,6,24,25^.

On the other hand, aromatic amines are very important in herbicide, dye, agrochemical and pesticide industries^26–28^. In addition, they are substrates for different intermediates such as diazonium salts, isocyanate, azo and amide molecules^29,30^. Recently, sodium borohydride, NaBH_4_, has been suggested as a new fuel source for supplying hydride ions to reduce nitroarene compounds to the corresponding amines under mild conditions. However, NaBH_4_ exhibits limited capability in the absence of additives^31–35^. Therefore, this reagent is usually used in the presence of metallic catalysts. Another important and conventional method for preparing the aromatic amines is the reduction of corresponding nitroarenes via catalytic hydrogenation^36,37^. The most metallic complexes and metallic nanoparticles applied for catalytic reduction of nitroarenes are based on Rh, Ru, Pt, Pd, Au, Cu, Ir and Ni^38–40^. Some of recently developed catalytic systems in this matter are Fe_3_O_4_/Ni MNPs^41^, Rh–Fe_3_O_4_ heterodimer^42^, Cu/Fe_2_O_4_–G^43^, graphene-Fe_3_O_4_^44^, Au–GO^45^, Fe_3_O_4_@GO^46^, Ag@Ni^47^ and NiFe_2_O_4_@Cu^48^. However, the homogeneous protocols for reduction of nitroarenes have different disadvantages such as separation of catalyst and product. Therefore, it is still necessary to design an effective catalytic system resolving these issues.

**Figure 1:**
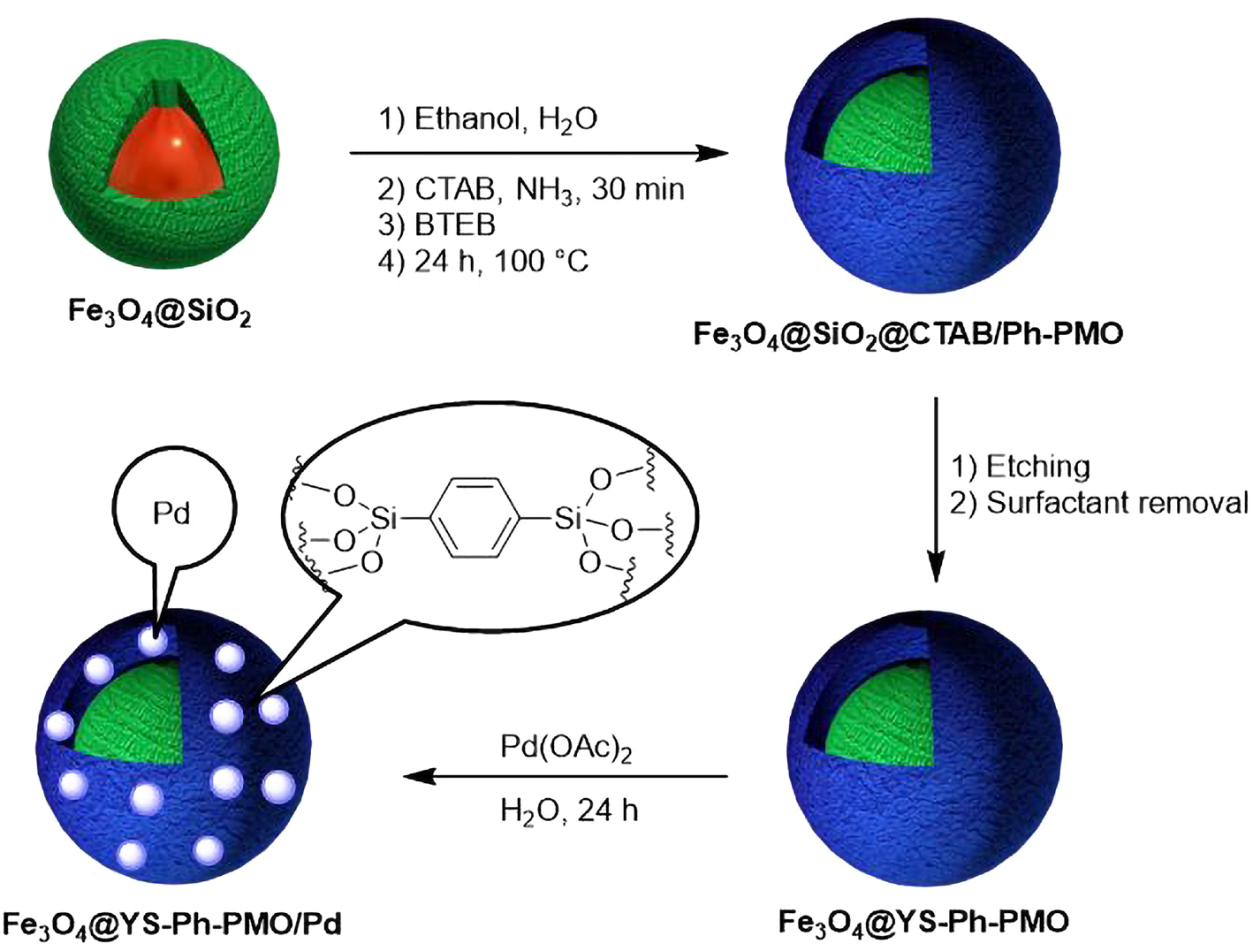
Preparation of Fe_3_O_4_@YS-Ph-PMO/Pd.

According to the advantages of Mag@YS-PMO-based nanocatalysts as well as the importance of reduction of nitroarenes in green chemistry, in continuation of our previous works^49–51^, herein, a YS nanomaterial with magnetite core and PMO shell-supported palladium species (Fe_3_O_4_@YS-Ph-PMO/Pd) is synthesized through sol–gel mediated hydrolysis and co-condensation of 1,4-bis(triethoxysilyl)benzene (BTEB) around Fe_3_O_4_@SiO_2_ cores using CTAB surfactant followed by treatment with palladium acetate (Fig. 1). Furthermore, the physiochemical properties of the synthesized Fe_3_O_4_@YS-Ph-PMO/Pd nanocatalyst were studied by using FT-IR, TGA, PXRD, SEM, TEM and VSM techniques. After characterization, the catalytic efficiency of this magnetic nanomaterial was investigated in the green reduction of nitroarenes under moderate conditions.

## Experimental

### General

All chemicals and reagents such as iron (II) chloride tetrahydrate, iron (III) chloride hexahydrate, ammonia, 1,4-bis(triethoxysilyl)benzene, NaBH_4_, cetyltrimethylammonium bromide (CTAB) and all applied nitroarenes and alcohols were purchased from Fluka, Merck and Aldrich companies. Solvents were dried and purified following standard procedures. The characterization of the materials was conducted using instruments previously reported^52,53^. The purity determination of the products and reaction monitoring were carried out by using TLC on silica gel polygram SILG/UV 254 plates.

### Preparation of Fe_3_O_4_@YS-Ph-PMO

For the preparation of Fe_3_O_4_@YS-Ph-PMO nanomaterial, firstly, Fe_3_O_4_ and silica coated magnetite nanoparticles (Fe_3_O_4_@SiO_2_) were synthesized according to known procedures^23^. Then, 100 mg of Fe_3_O_4_@SiO_2_ microspheres were added into a flask containing EtOH (60 mL) and H_2_O (80 mL). This mixture was homogenized for 25 min under ultrasound irradiations. Next, CTAB (140 mg) and NH_3_ (25%, 2 mL) were added while stirring at RT for 30 min. After that, BTEB (0.3 mL) was added and it was further stirred at RT for 2 h. This mixture was heated statically at 100 °C for 24 h. The obtained Fe_3_O_4_@SiO_2_@CTAB@Ph-PMO product was dispersed in a basic solution (H_2_O:Na_2_CO_3_, 80:4). The resulting mixture was heated to 50 °C for 4 h to eliminate the SiO_2_ shell. The CTAB surfactant was successfully eliminated by refluxing of as-made material in an acidic EtOH solution (EtOH:HCl 100:2). The final product was dried at 70 °C for 12 h and denoted as Fe_3_O_4_@YS-Ph-PMO.

### Preparation of Fe_3_O_4_@YS-Ph-PMO/Pd

For this, Fe_3_O_4_@YS-Ph-PMO (0.4 g) was added to an aqueous solution of palladium acetate (0.05 M, 14 mL). Then, this mixture was stirred at RT for 24 h. The product was magnetically collected, washed completely with H_2_O, dried at 70 °C for 7 h and denoted as Fe_3_O_4_@YS-Ph-PMO/Pd.

### Procedure for the reduction of nitrobenzenes

For this, nitrobenzene (1 mmol), Fe_3_O_4_@YS-Ph-PMO/Pd (0.9 mol %) and H_2_O (10 mL) were added into a reaction vessel. Then, an aqueous solution of NaBH_4_ (3 mmol) was added while stirring at RT. After completion of reaction, the catalyst was magnetically separated and the amine product was obtained after extraction with EtOAc and evaporation of solvent. The IR and NMR spectra of prepared aminobenzenes are available in the supporting information.

## Results and discussion

The preparation of Fe_3_O_4_@YS-Ph-PMO/Pd nanocatayst with yolk-shell structure is shown in Fig. 1. At first, the magnetic silica (Fe_3_O_4_@SiO_2_) was prepared by chemical modification of Fe_3_O_4_ nanoparticles with tetramethoxysilane (TMOS). Then, 1,4-bis(triethoxysilyl)benzene (BTEB) was hydrolyzed and co-condensed on the Fe_3_O_4_@SiO_2_ spheres in the presence of CTAB surfactant in a basic ammonia–water–ethanol solution through sol–gel process. Then, the silica layer was removed in an aqueous solution of Na_2_CO_3_ through an etching process. After that, the CTAB surfactant was removed by a Soxhlet apparatus to give a yolk-shell structured material called Fe_3_O_4_@YS-Ph-PMO. The resulting material was then treated with a sub-stoichiometric amount of Pd(OAc)_2_ in water to produce the Fe_3_O_4_@YS-Ph-PMO/Pd nanocatalyst.

**Figure 2:**
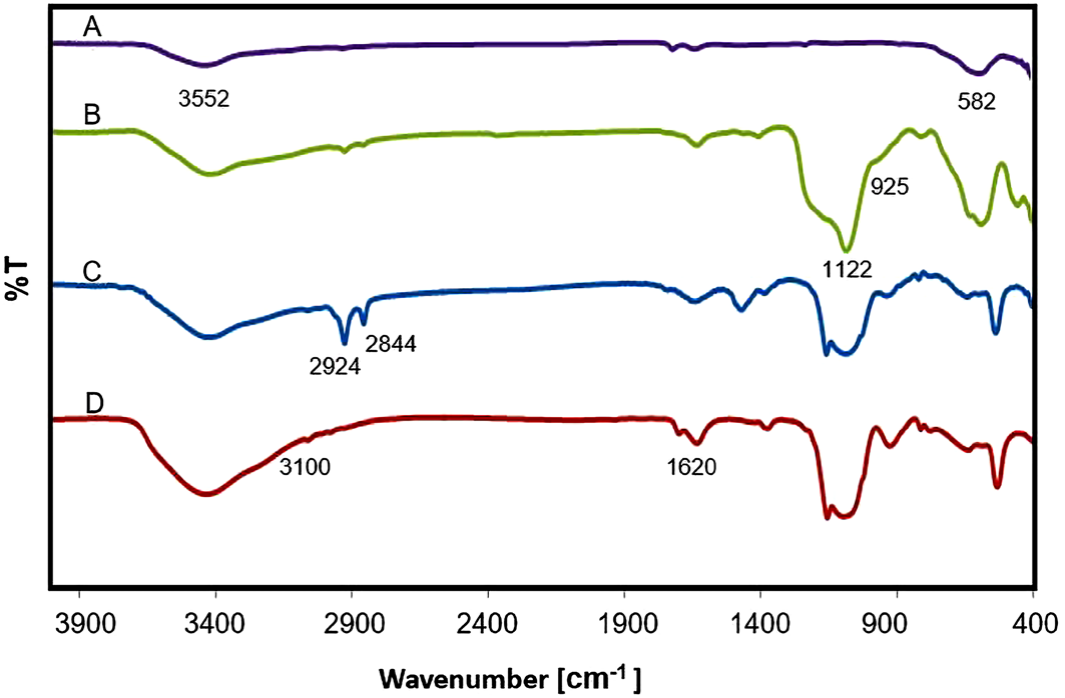
FT-IR spectra of (**A**) Fe_3_O_4_, (**B**) Fe_3_O_4_@SiO_2_, (**C**) Fe_3_O_4_@SiO_2_@CTAB@Ph-PMO and (**D**) Fe_3_O_4_@YS-Ph-PMO/Pd.

The Fourier transform infrared (FT-IR) spectra of prepared materials are shown in Fig. 2. For all samples, the Fe–O bond is cleared at 582 cm^−1^. The band about 3552 cm^−1^ is due to the O–H bonds of material surface. For Fe_3_O_4_@SiO_2_, Fe_3_O_4_@SiO_2_@CTAB@YS-Ph-PMO and Fe_3_O_4_@YS-Ph-PMO/Pd, the peaks at 1122 and 925 cm^−1^ are attributed to Si–O-Si bonds. The bands at 2924 and 2844 cm^−1^ are related to the C–H vibrations of CTAB surfactant (Fig. 2C). Interestingly, for Fe_3_O_4_@YS-Ph-PMO/Pd, the latter peaks are eliminated (Fig. 2D), indicating the successful removal of CTAB surfactant during extraction process. For Fe_3_O_4_@SiO_2_@CTAB@YS-Ph-PMO and Fe_3_O_4_@YS-Ph-PMO/Pd, the peaks observed at 3100 and 1620 cm^−1^ are, respectively, correspond to C–H and C=C vibrations of phenyl rings. These confirm the successful formation of Ph-PMO shell on magnetite NPs. Notably, the Fe–O absorption peaks of Fe_3_O_4_ and Fe_3_O_4_@SiO_2_ exhibited a slight red shift compared to Fe_3_O_4_@SiO_2_@CTAB@Ph-PMO and Fe_3_O_4_@YS-Ph-PMO/Pd nanomaterials. This shift is in line with the Bouguer-Beer-Lambert (BBL) law, where spectral positions correspond to sample thickness and absorbing entity concentration. The presence of silica, CTAB, and PMO layers on Fe_3_O_4_ nanoparticles contributes to this red shift, indicating a modified chemical environment surrounding the nanoparticles^54–56^. Moreover, an increase in layer thickness enhances this effect^57–59^.

**Figure 3:**
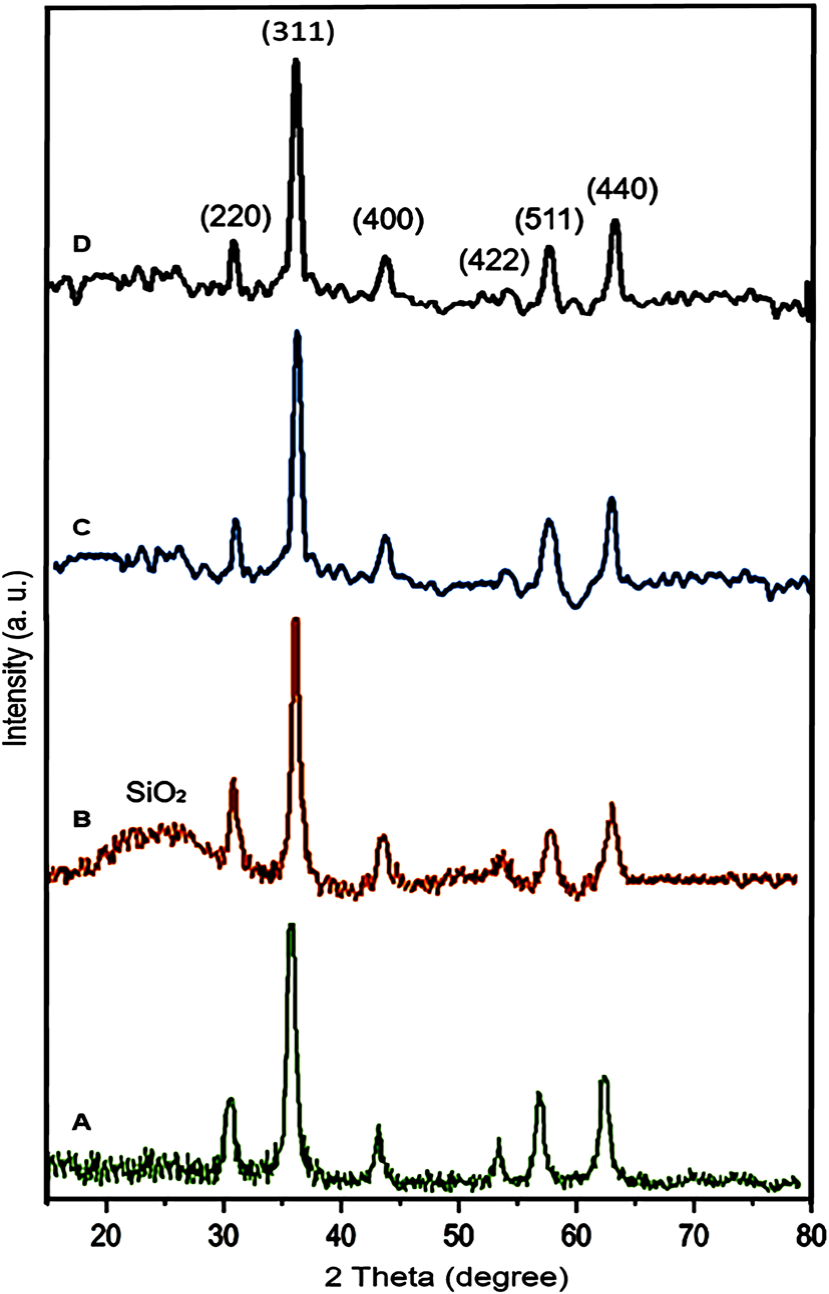
PXRD patterns of (**A**) Fe_3_O_4_, (**B**) Fe_3_O_4_@SiO_2_, (**C**) Fe_3_O_4_@YS-Ph-PMO and (**D**) Fe_3_O_4_@YS-Ph-PMO/Pd.

Figure 3 shows the wide-angle powder X-ray diffraction (PXRD) patterns of Fe_3_O_4_, Fe_3_O_4_@SiO_2_, Fe_3_O_4_@YS-Ph-PMO and Fe_3_O_4_@YS-Ph-PMO/Pd nanomaterials. The PXRD patterns of all the materials showed six sharp peaks at 2θ of 30.38, 35.65, 43.34, 53.9, 57.40 and 62.98°, corresponding to the *Miller indices* of 220, 311, 400, 422, 511, and 440, respectively, which is completely matched with that of the Fe_3_O_4_ standard sample (JCPDS file No. 19-0629)^60^. This finding proves that the crystalline structure of the magnetic iron oxide NPs is preserved during the modification processes. Additionally, a continuous decrease in the intensity of Fe_3_O_4_ peaks was observed, indicating the successful modification of magnetic iron oxide with the organic and inorganic species. After coating with silica, a new and broad peak at 2θ = 20–25 appeared, which is attributed to the presence of amorphous silica (Fig. 3B). This peak disappeared in both Fe_3_O_4_@YS-Ph-PMO and Fe_3_O_4_@YS-Ph-PMO/Pd (Fig. 3C and D), confirming the successful removal of the silica layer through the etching process.

**Figure 4:**
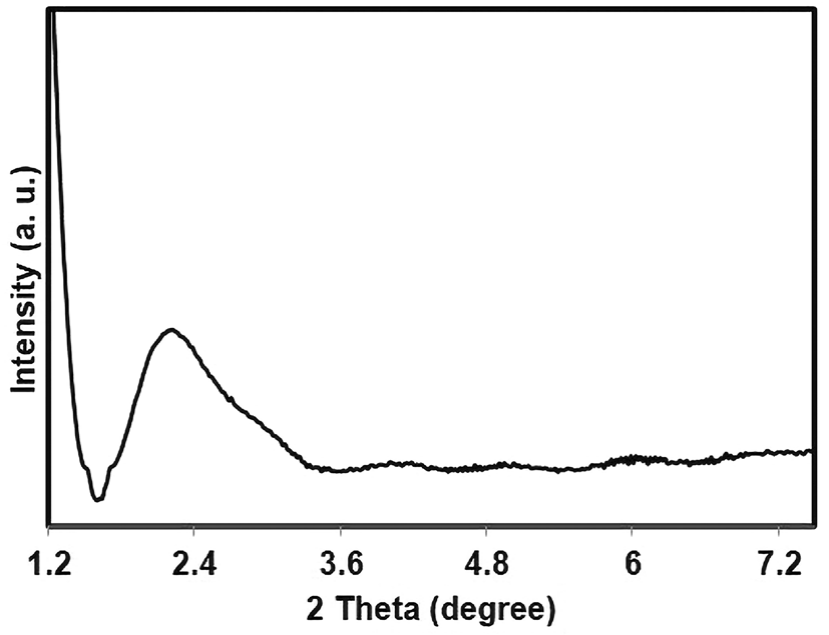
Low-angle PXRD pattern of Fe_3_O_4_@YS-Ph-PMO/Pd.

The low-angle powder X-ray diffraction (PXRD) pattern of the Fe_3_O_4_@YS-Ph-PMO/Pd is shown in Fig. 4. This illustrates a broad peak at 2θ = 2.2°, which is characteristic of ordered mesoporous structures. This pattern confirm well formation of Ph-PMO shell on magnetite NPs.

**Figure 5:**
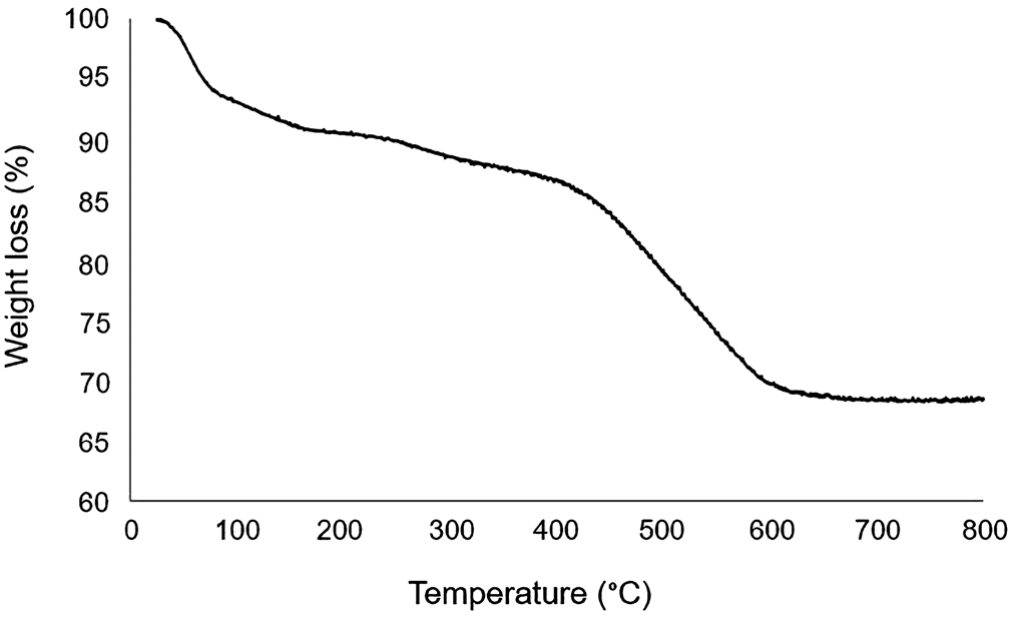
TGA of Fe_3_O_4_@YS-Ph-PMO/Pd.

Thermal stability of Fe_3_O_4_@YS-Ph-PMO/Pd was studied by using thermal gravimetric analysis (TGA) (Fig. 5). This showed a low weight loss at temperature below 100 °C, corresponding to removal of adsorbed solvents. The second weight loss observed between 100 to 450 °C is related to the elimination of the remaining CTAB surfactant. The main weight loss, cleared at 451–620 °C, is corresponded to the removal of organic (phenylene) moieties incorporated in the mesoporous shell. These data successfully prove the high thermal stability of the designed nanocatalyst.

**Figure 6:**
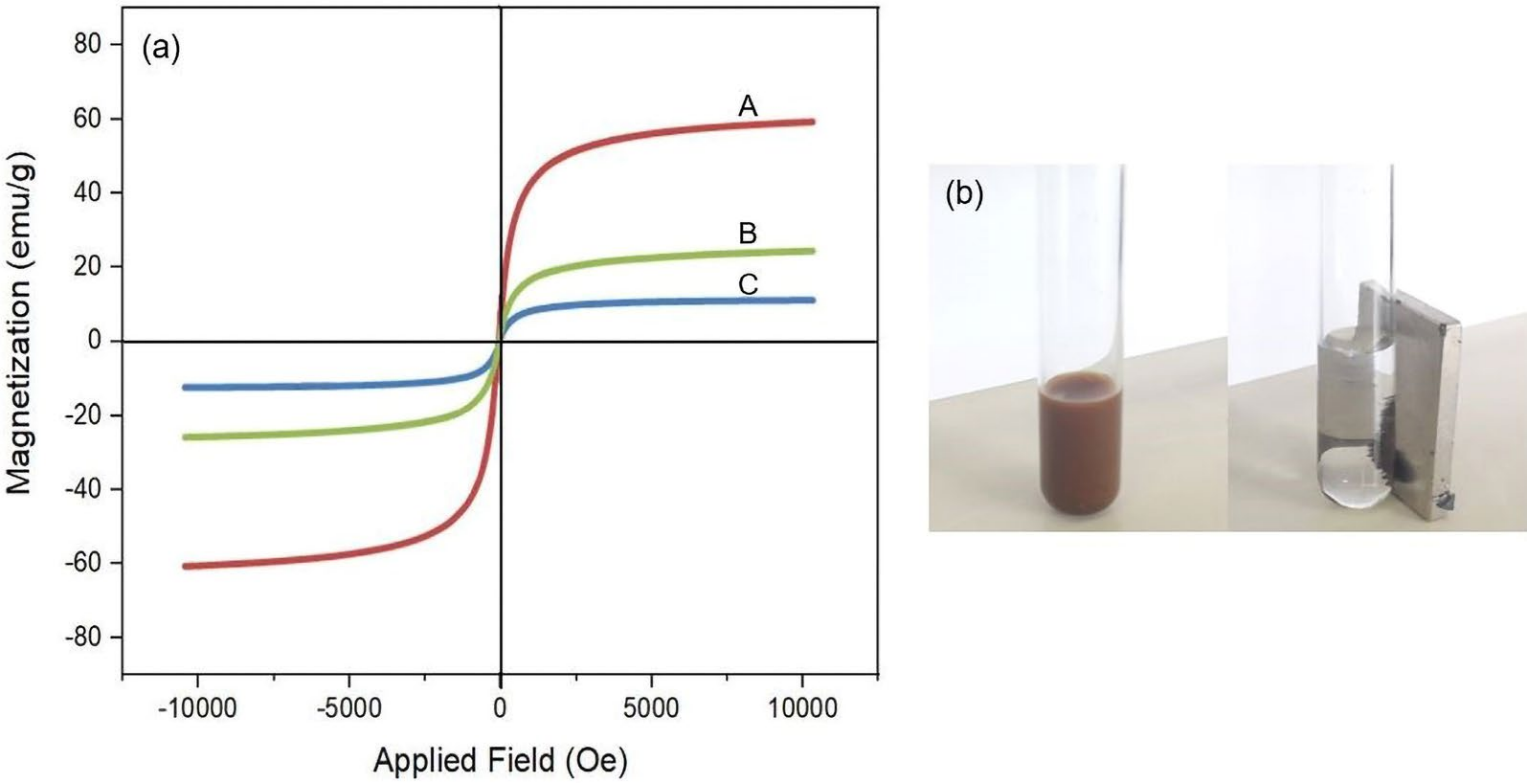
(**a**) VSM diagrams of Fe_3_O_4_ (A), Fe_3_O_4_@SiO_2_ (B) and Fe_3_O_4_@YS-Ph-PMO/Pd (C) nanomaterials and (**b**) magnetic separation ability of Fe_3_O_4_@YS-Ph-PMO/Pd nanocatalyst.

The magnetic properties of materials were investigated by vibrating sample magnetometer (VSM) (Fig. 6). The saturated magnetization values of the Fe_3_O_4_ (Fig. 6A), Fe_3_O_4_@SiO_2_ (Fig. 6B) and Fe_3_O_4_@YS-Ph-PMO/Pd (Fig. 6C) were found to be 65, 30 and 14 emu g^−1^, respectively. The decrease in magnetic properties following the modification processes is attributed to the well coating of SiO_2_ and PMO shells around Fe_3_O_4_ NPs. The magnetic separation capability of the Fe_3_O_4_@YS-Ph-PMO/Pd nanocomposite was also evaluated by introducing an external magnet near the reaction vessel, as depicted in Fig. 6b. As demonstrated, these nanomaterials can be efficiently collected by using an external magnet within seconds, confirming the high magnetic properties of the designed catalyst, rendering it readily recoverable.

**Figure 7:**
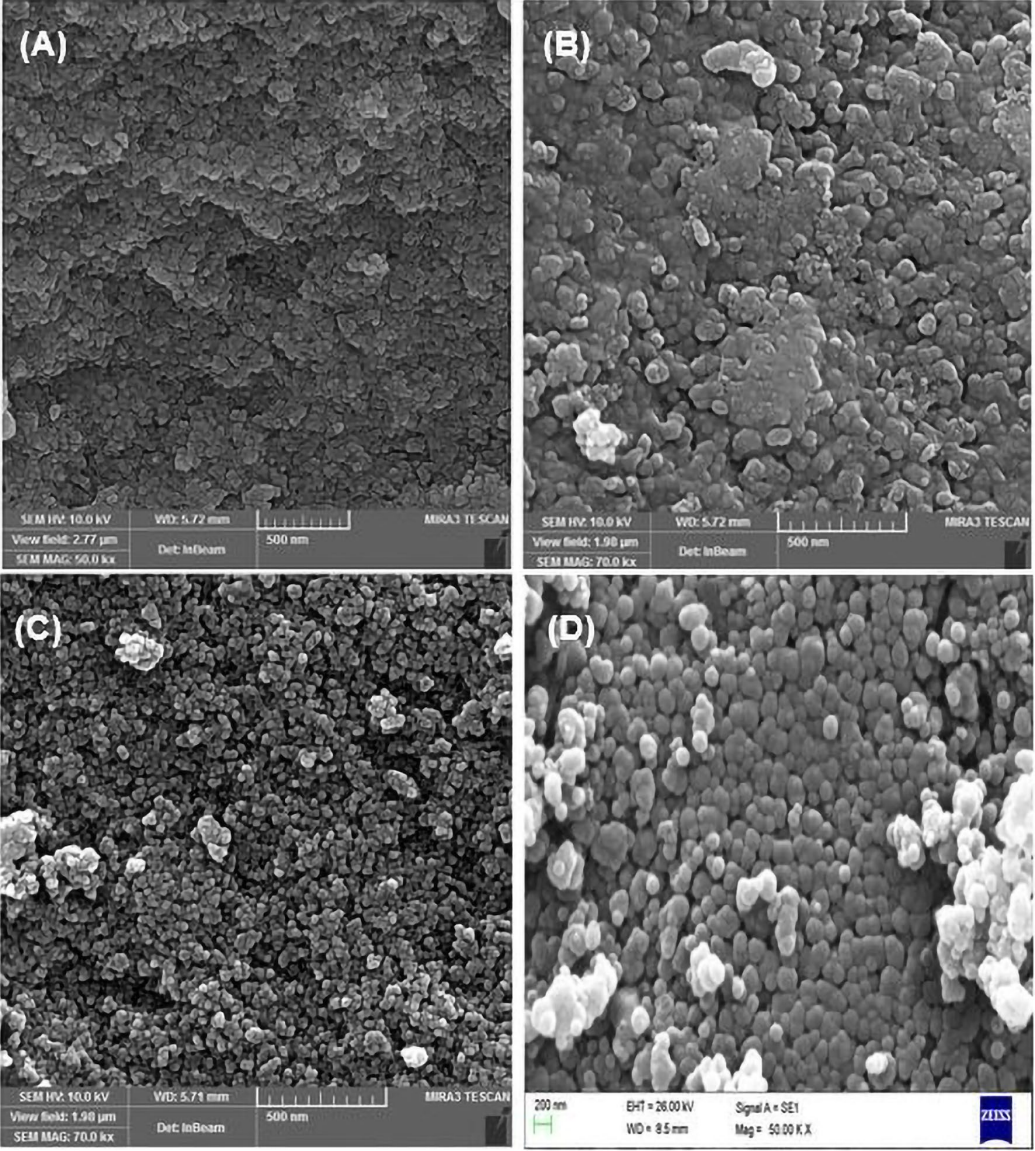
SEM images of (**A**) Fe_3_O_4_, (**B**) Fe_3_O_4_@SiO_2_, (**C**) Fe_3_O_4_@YS-Ph-PMO and (**D**) Fe_3_O_4_@YS-Ph-PMO/Pd.

The scanning electron microscopy (SEM) was performed to study the morphology of the particles at different steps of nanocatalyst preparation. According to this analysis, a uniform spherical morphology was observed for all prepared nanomaterials with an increase in size observed at each step (Fig. 7). The progressive increase in particle size after each step confirms the successful formation of the shell and modification of magnetite nanoparticles, as outlined in Fig. 1.

**Figure 8:**
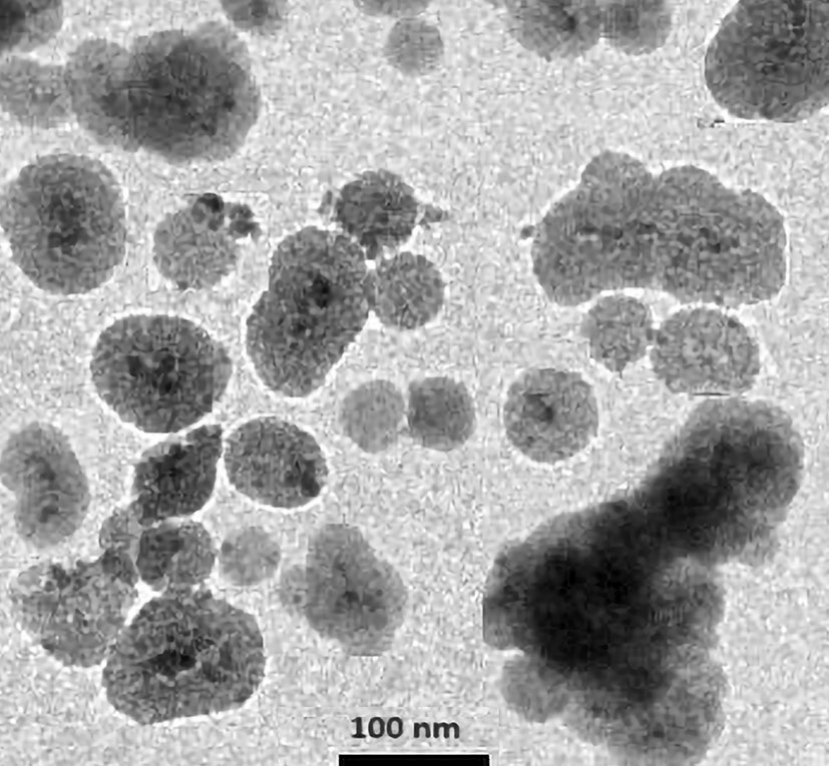
TEM image of the Fe_3_O_4_@YS-Ph-PMO/Pd nanocatalyst.

The TEM image of the Fe_3_O_4_@YS-Ph-PMO/Pd nanocatalyst showed spherical particles with a black core (magnetite NPs) and a grey shell (PMO layer) for the designed nanocomposite (Fig. 8). Notably, recently, the similar TEM images have been reported for a number of yolk-shell structured magnetic nanocomposites^61,62^.

The Fe_3_O_4_@YS-Ph-PMO/Pd nanocatalyst was then employed in the reduction of nitroarenes in the presence of NaBH_4_ (Table 1). The effect of different parameters was studied in the reduction of nitrobenzene as a reaction model. As shown, in the absence of catalyst, along with 3 mmol of NaBH_4_, no reduction was observed (Table 1, entry 1). However, after addition of the catalyst, the reaction proceeded successfully, and the highest yield achieved using 0.9 mol% of the Fe_3_O_4_@YS-Ph-PMO/Pd (Table 1, entry 3). Next, the effect of different solvents including methanol, ethanol and water was tested. As depicted in Table 1, the use of MeOH, EtOH, aqueous methanol or ethanol, as well as solvent-free media, resulted in low to moderate yields (Table 1, entries 5–9). Pleasantly, in aqueous media at RT, the reaction was completed and excellent yield of aniline was obtained. To investigate the neat effect of Pd, the efficiency of Fe_3_O_4_@YS-Ph-PMO/Pd was compared with Fe_3_O_4_ and Fe_3_O_4_@YS-Ph-PMO nanomaterials. This demonstrated that when using Pd-free Fe_3_O_4_ and Fe_3_O_4_@Ph-PMO materials, the reaction did not progress, and only a trace conversion (< 4%) was observed (Table 1, entries 10 and 11). This confirms that the reduction process is catalyzed by supported palladium species. Based on the aforementioned results, NaBH_4_ (3 mmol), H_2_O solvent (3 mL), Fe_3_O_4_@YS-Ph-PMO/Pd catalyst (0.9 mol %) and RT were chosen as the optimum conditions (Table 1, entry 3).

**Table 1:** Reduction of nitrobenzene in the presence of Fe_3_O_4_@YS-Ph-PMO/Pd^a^.

Entry	Catalyst	Catalyst loading (mol %)	Time (min)	Solvent	Temperature	Yield (%.)^b^
1	–	–	300	H_2_O	Reflux	N. R.
2	Fe_3_O_4_@YS-Ph-PMO/Pd	0.6	10	H_2_O	RT	78
3	Fe_3_O_4_@YS-Ph-PMO/Pd	0.9	10	H_2_O	RT	96
4	Fe_3_O_4_@YS-Ph-PMO/Pd	1.2	10	H_2_O	RT	96
5	Fe_3_O_4_@YS-Ph-PMO/Pd	0.9	10	Solvent-free	RT	63
6	Fe_3_O_4_@YS-Ph-PMO/Pd	0.9	10	MeOH	RT	57
7	Fe_3_O_4_@YS-Ph-PMO/Pd	0.9	10	MeOH/H_2_O	RT	75
8	Fe_3_O_4_@YS-Ph-PMO/Pd	0.9	10	EtOH	RT	68
9	Fe_3_O_4_@YS-Ph-PMO/Pd	0.9	10	EtOH/H_2_O	RT	92
10	Fe_3_O_4_	0.03 g	10	H_2_O	RT	Trace
11	Fe_3_O_4_@YS-Ph-PMO	0.03 g	10	H_2_O	RT	Trace

^a^Nitrobenzene (1 mmol), NaBH_4_ (3 mmol), Fe_3_O_4_@YS-Ph-PMO/Pd nanocatalyst and solvent (3 mL).

^b^Isolated yields.

The efficiency and the scope of the Fe_3_O_4_@YS-Ph-PMO/Pd nanocatalyst were next studied using a variety of nitrobenzenes (Table 2). As shown, all nitroarenes deliver corresponding anilines in high yields. Importantly, in all cases, it was found that the aniline derivatives were the only products of the reactions. These results demonstrate high selectivity of the designed catalytic system.

**Table 2:** Reduction of different nitrobenzenes using Fe_3_O_4_@YS-Ph-PMO/Pd catalyst^a^.


Entry	Substrate	Product	Time (min)	Yield (%)^b^
1			10	96
2			12	96
3			12	94
4			15	93
5			14	95
6			15	91
7			18	92

^a^Nitrobenzene (1 mmol), NaBH_4_ (3 mmol), H_2_O (3 mL) and catalyst (0.9 mol%).

^b^Isolated yields.

**Figure 9:**
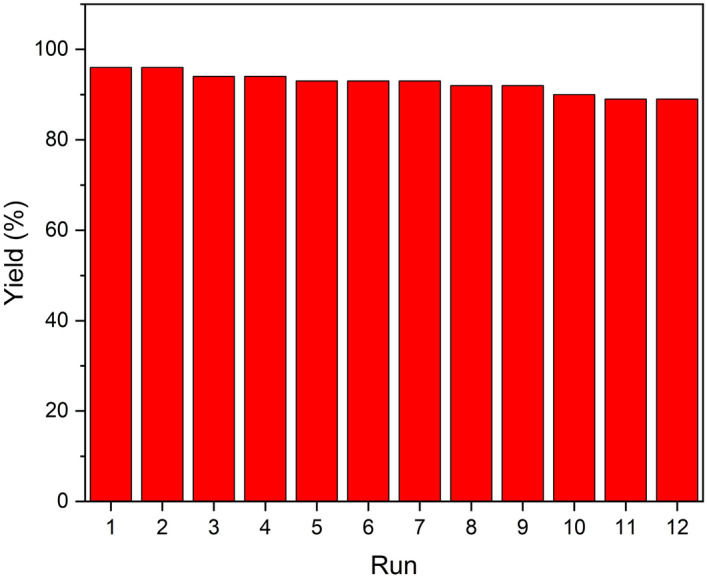
Recoverability and reusability of the Fe_3_O_4_@YS-Ph-PMO/Pd nanocatalyst in the reduction of nitrobenzene.

Recoverability and reusability of the Fe_3_O_4_@YS-Ph-PMO/Pd nanocatalyst in the reduction of nitrobenzene were also examined. For this, after completion of the reaction, the Fe_3_O_4_@YS-Ph-PMO/Pd catalyst was magnetically separated, washed and reused in the next run. It was found that the Fe_3_O_4_@YS-Ph-PMO/Pd catalyst can be recovered and reused for at least 11 times with no important decrease in efficiency (Fig. 9).

**Figure 10:**
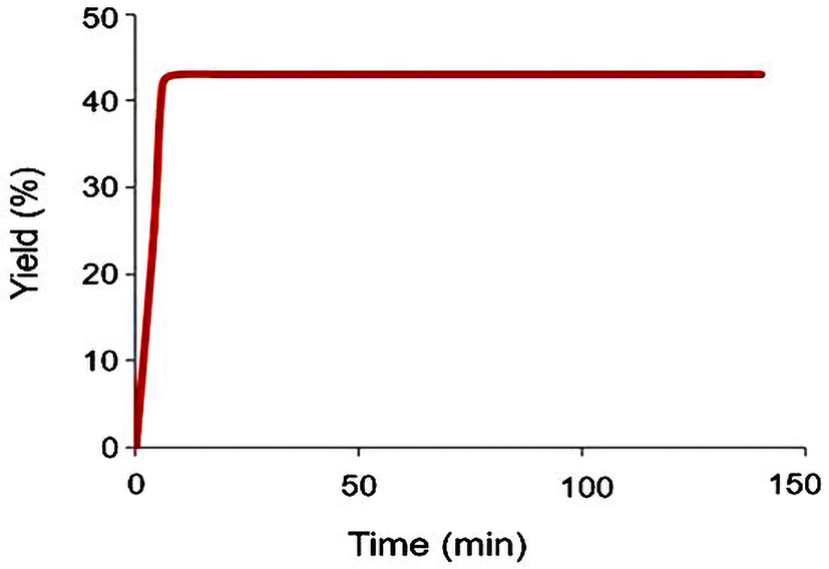
Result of the leaching test in the reduction of nitrobenzene in the presence of Fe_3_O_4_@YS-Ph-PMO/Pd nanocatalyst.

A leaching experiment was then accomplished on the model reaction to investigate the leaching behavior of the palladium species under applied conditions. For this, after a conversion of about 40%, the Fe_3_O_4_@YS-Ph-PMO/Pd catalyst was magnetically separated and the residue mixture was allowed to progress under the optimized conditions. Notably, after 140 min, no further product was observed indicating that the removal of the catalyst resulted in a complete stop of the conversion of nitrobenzene to aniline (Fig. 10). These results confirm no leaching of palladium species and also demonstrate the high stability of the designed catalyst under applied conditions.

The catalytic performance of Fe_3_O_4_@YS-Ph-PMO/Pd was also compared with former heterogeneous catalysts in the reduction of nitrobenzene (Table 3). The result showed that the efficiency of Fe_3_O_4_@YS-Ph-PMO/Pd is much better than previous catalytic systems, particularly in terms of reaction temperature and recovery times.

**Table 3:** Comparison of catalytic activity of Fe_3_O_4_@YS-Ph-PMO/Pd nanocatalyst with previously reported catalytic systems in the reduction of nitrobenzene.

Entry	Catalyst	Conditions	Recovery times	^Refs.^
1^a^	γ-Fe_2_O_3_@SiO_2_–Pt@mSiO_2_	Hydrogen gas, EtOH, 40 °C	5	^63^
2^b^	γ-Fe_2_O_3_-MNAs	MeNHNH_2_, EtOH, 60 °C	7	^64^
3^c^	RHPrNH_2_@Ag	NaBH_4_, H_2_O, Reflux	4	^65^
4^d^	Fe_3_O_4_@nSiO_2_@mSiO_2_/Pr-Imi-NH_2_·Ag	NaBH_4_, H_2_O, 95 °C	4	^66^
5	Pd/SBA-15	Hydrogen gas, decane, EtOH, 313 K	5	^67^
6	Pt–Pd/PMO-SBA-15	Hydrogen gas, EtOH, 60 °C	5	^68^
7	Fe_3_O_4_@YS-Ph-PMO/Pd	NaBH_4_, H_2_O, RT	11	This work

^a^mSiO_2_, mesoporous silica.

^b^MNAs, mesoporous nanoparticle assemblies.

^c^RHPrNH_2_, rice husk-SiO_2_ aminopropylsilane.

^d^Pr-Imi, propylimidazolium.

## Conclusion

In this work, the preparation, characterization and application of a new nanocatalyst named Fe_3_O_4_@YS-Ph-PMO/Pd were developed. The FT-IR and TG analyses clearly demonstrated high stability and well incorporation/immobilization of expected organic and inorganic moieties onto/into prepared nanomaterial. The SEM and TEM images showed a spherical morphology for the designed catalyst. The VSM analysis confirmed well-magnetic properties of the catalyst. The wide-angle PXRD analysis confirmed high stability of crystalline structure of magnetite NPs during catalyst preparation steps. The Fe_3_O_4_@YS-Ph-PMO/Pd was powerfully used in the reduction of nitrobenzenes giving corresponding anilines in high yield and selectivity. The leaching and recovery experiments confirmed that the designed catalyst operate in a heterogeneous manner. Other advantages of this methodology were the use of water as green solvent, performing reactions at RT, short reaction times, clean conditions, as well as high recoverability, durability and stability of the designed catalyst. Some applications of this catalytic system in other organic processes are underway in our laboratory.

### Supplementary Information

Supplementary Information.

## Data Availability

All data and materials are included in the manuscript.
