# Supplementary material for: Magnetic yolk-shell structured periodic mesoporous organosilica supported palladium as a powerful and highly recoverable nanocatalyst for the reduction of nitrobenzenes
Source: Sci Rep. 2024 Jul 15;14:16262. doi: 10.1038/s41598-024-66883-4 (PMC11251011; doi:10.1038/s41598-024-66883-4)
Supplement: Supplementary file 1 — Supplementary Information. [file 41598_2024_66883_MOESM1_ESM.pdf]

Supporting Information for:

Magnetic yolk-shell structured periodic mesoporous organosilica supported palladium as a powerful and highly recoverable nanocatalyst for the reduction of nitrobenzenes

Meysam Norouzi, Dawood Elhamifar* and Shiva Kargar

Department of Chemistry, Yasouj University, Yasouj, 75918-74831, Iran

Email: d.elhamifar@yu.ac.ir

Contents

FT-IR, ^{1}H NMR and ^{13}C NMR spectra of aminobenzenes

Aniline (2a)

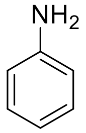

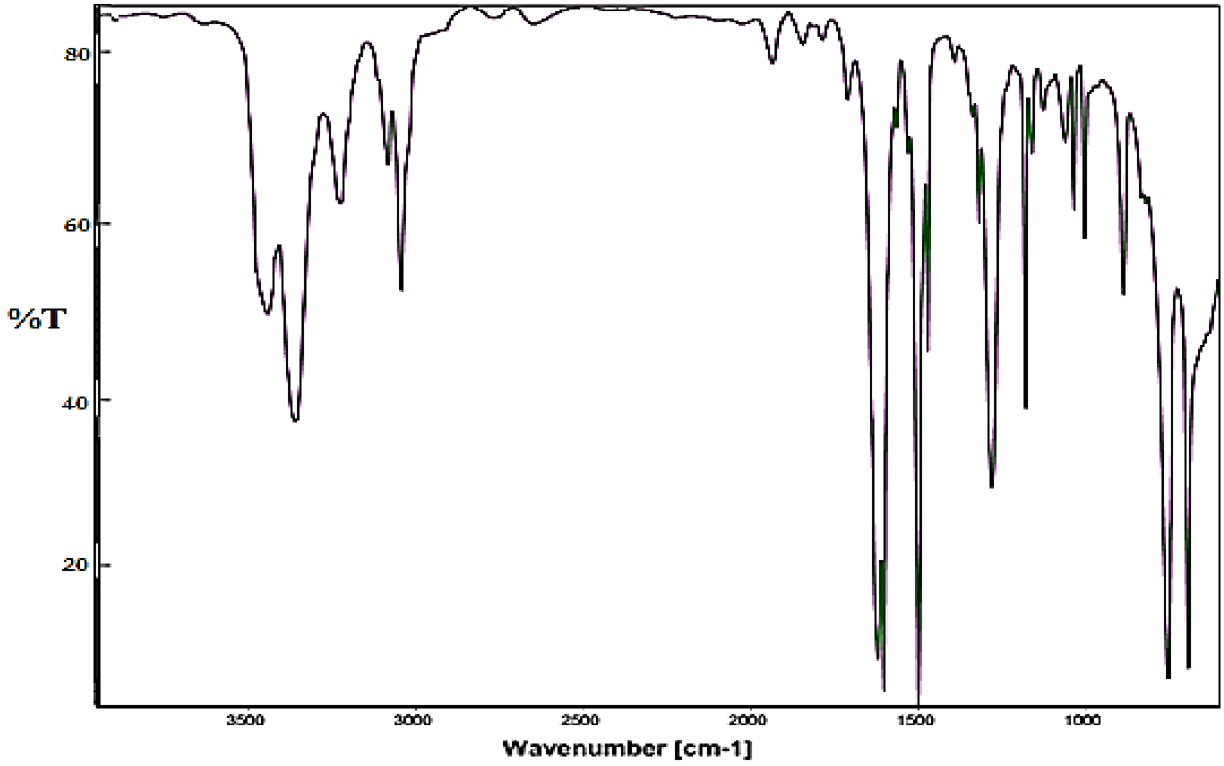

FT-IR spectrum of compound **2a**

4-Aminobenzyl alcohol (2b)

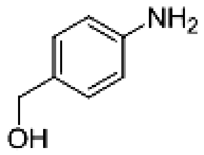

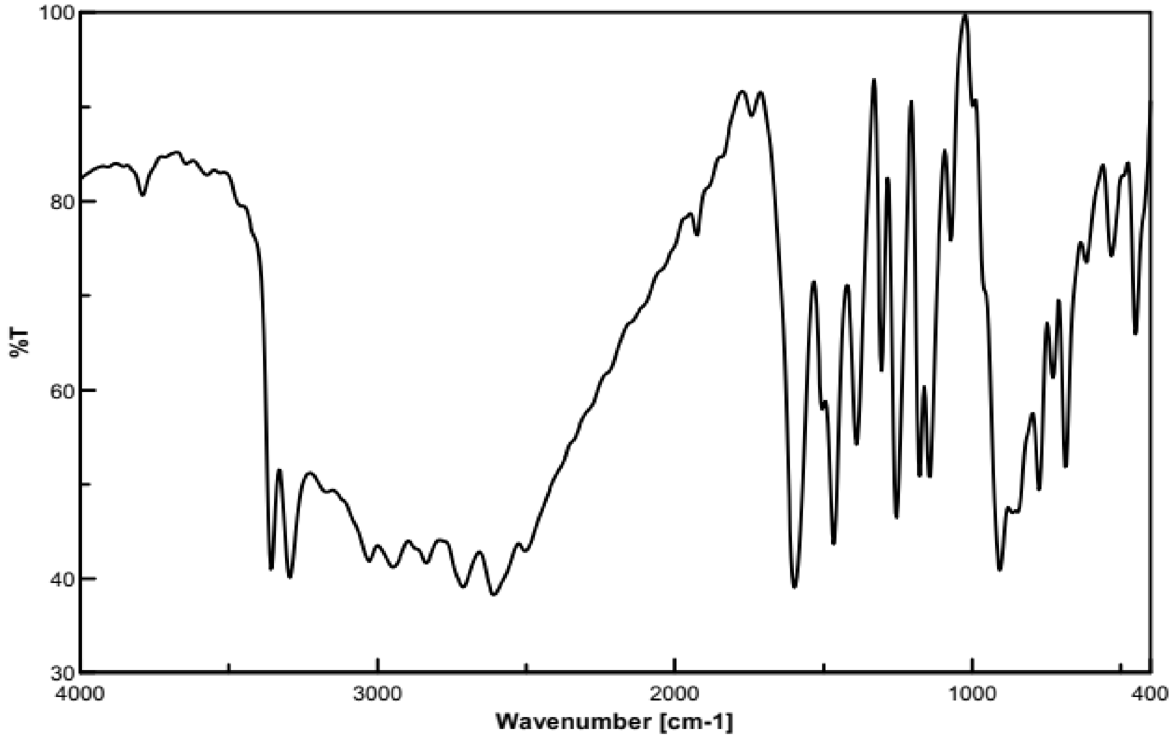

FT-IR spectrum of compound **2b**

p-Toluidine (2c)

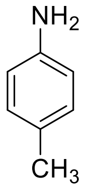

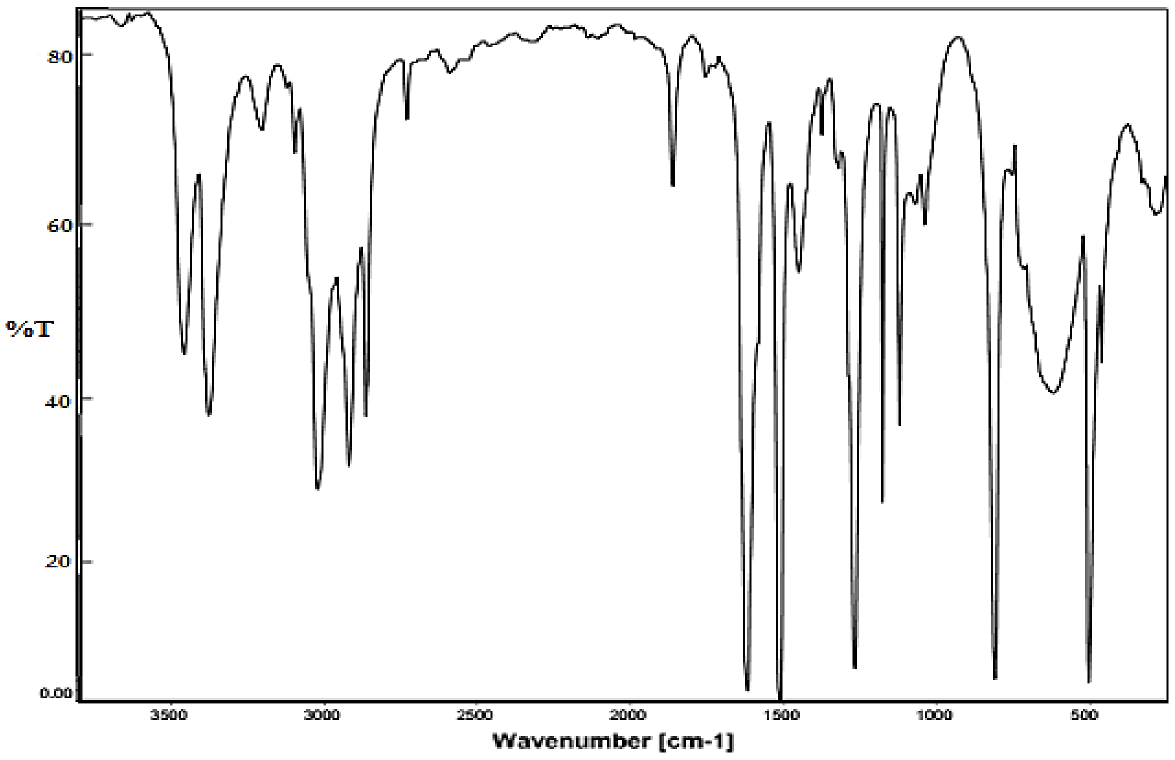

FT-IR spectrum of compound **2c**

4-Chloroaniline (2d)

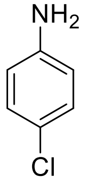

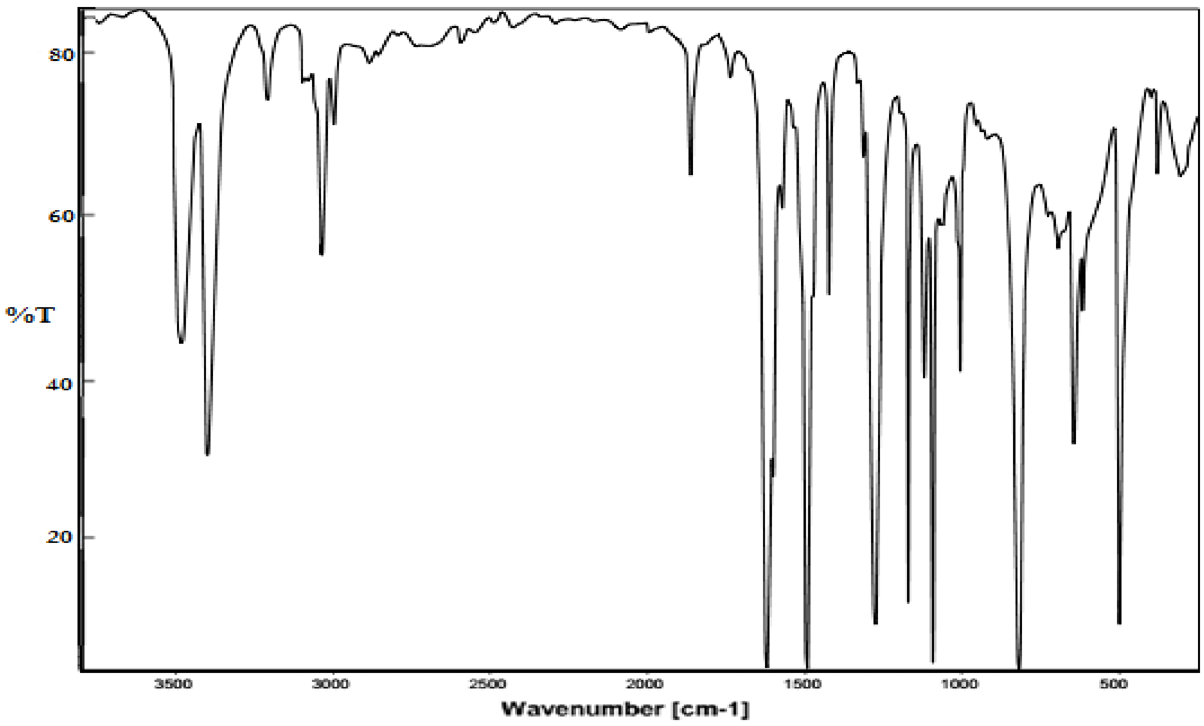

FT-IR spectrum of compound **2d**

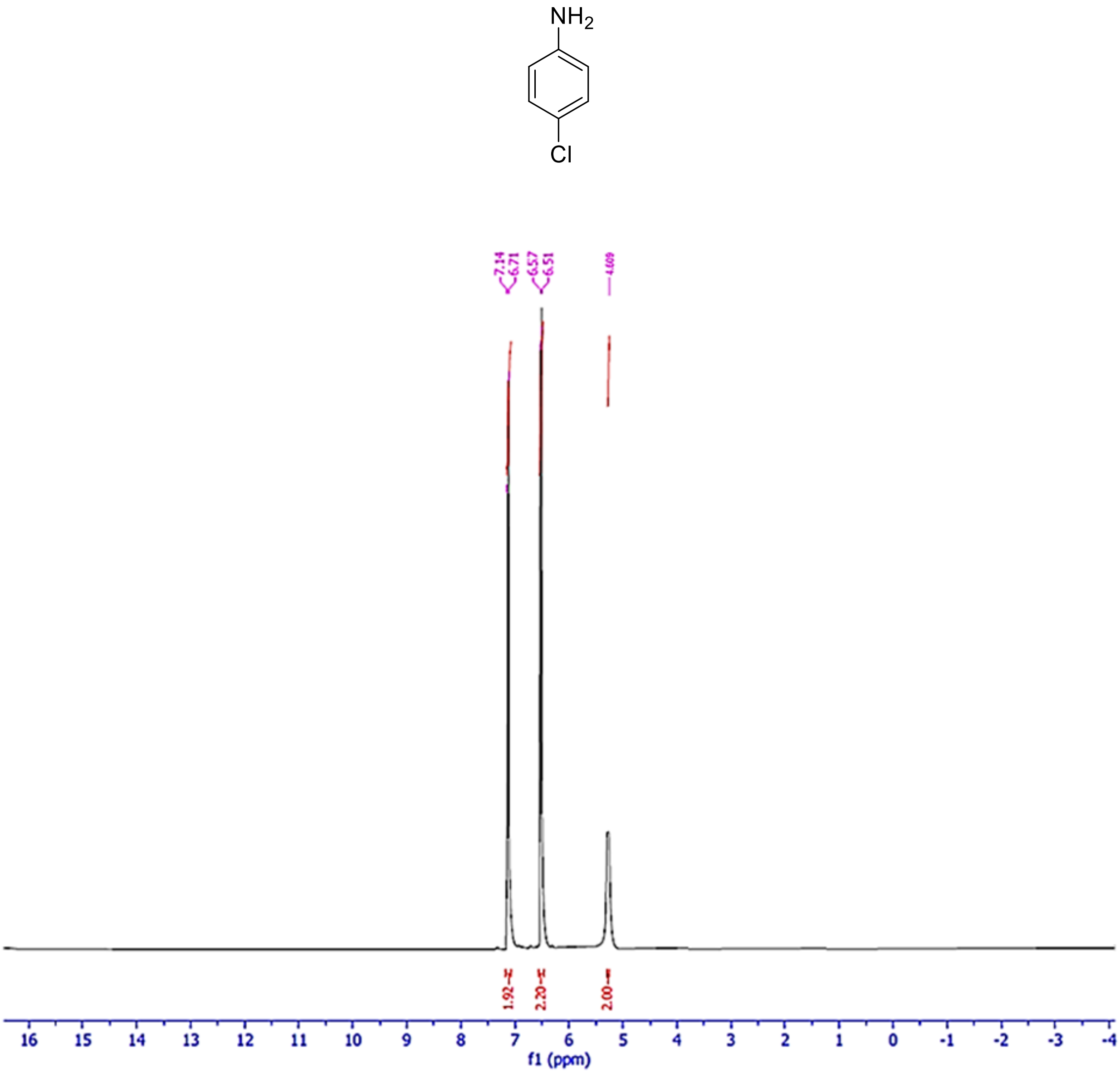

^{1}H NMR spectrum of compound **2d**

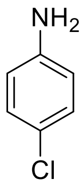

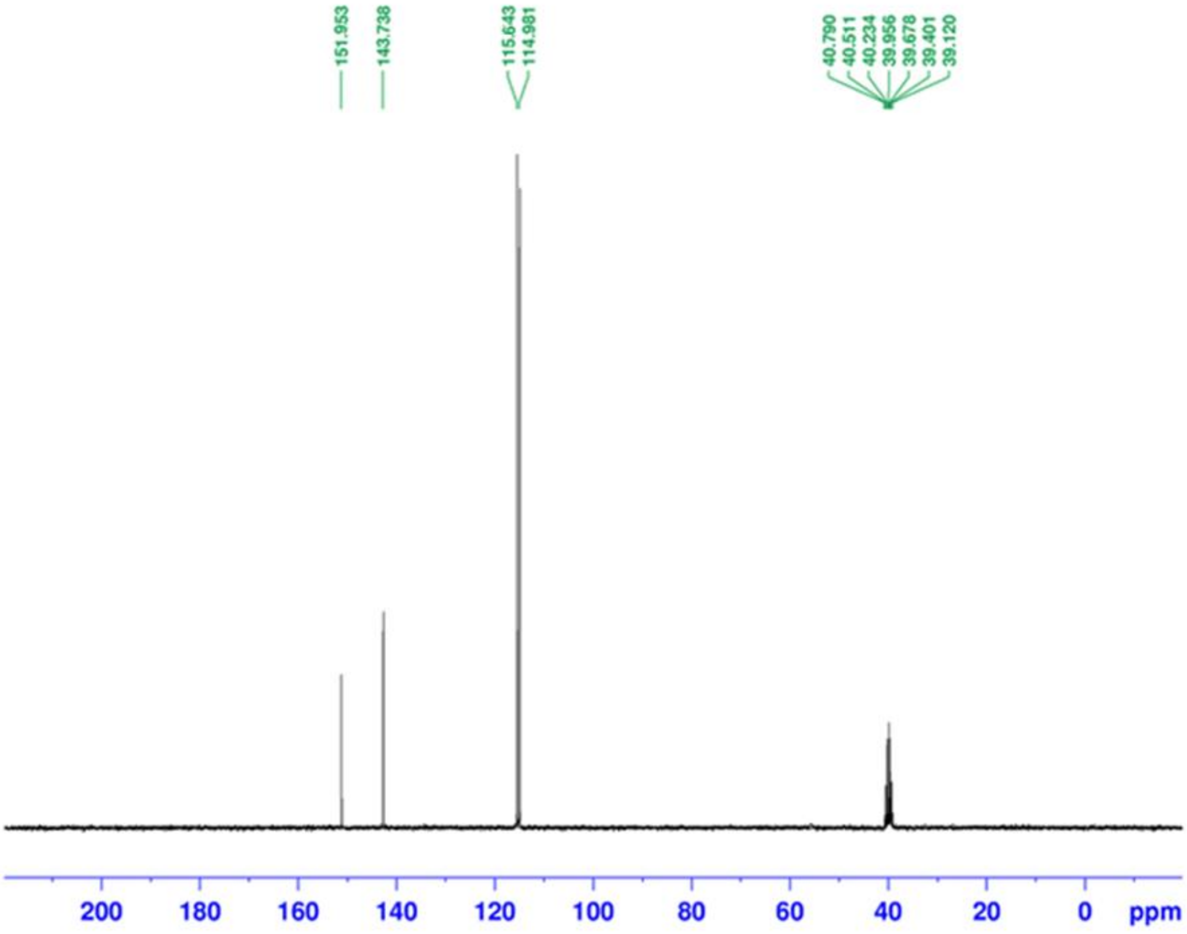

^{13}C NMR spectrum of compound **2d**

Benzene-1,3-diamine (2e)

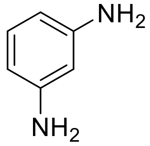

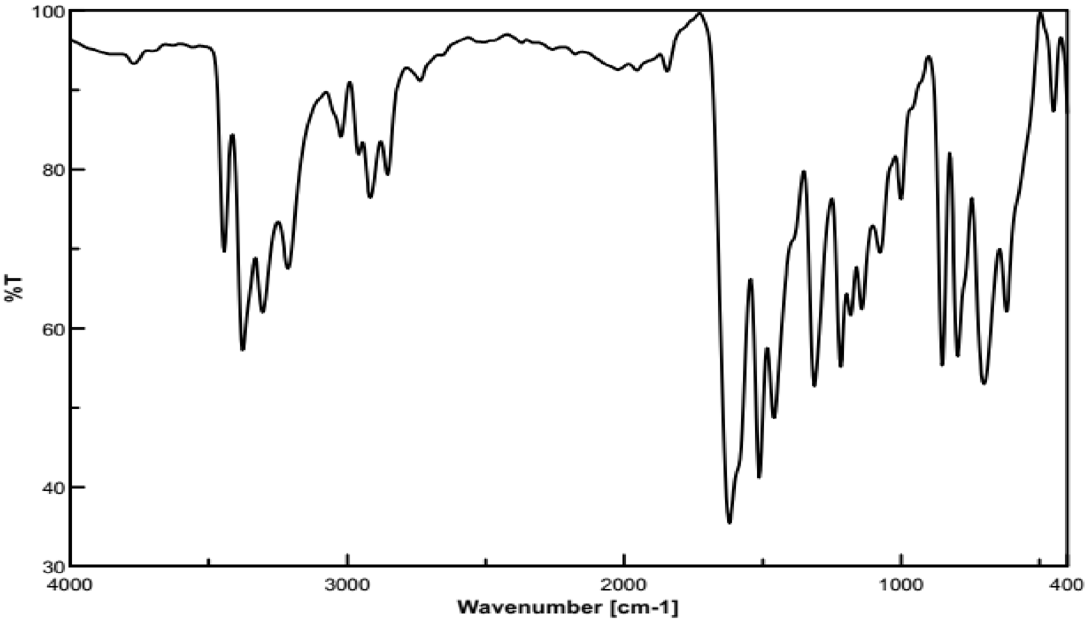

FT-IR spectrum of compound **2e**

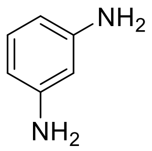

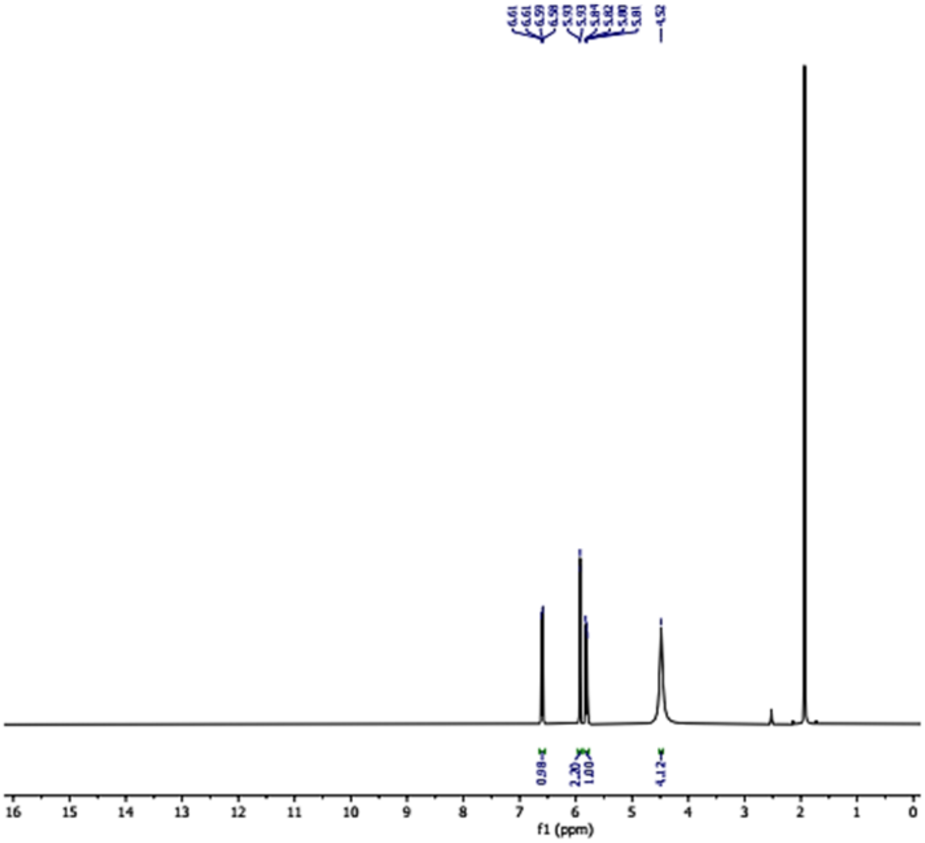

^{1}H NMR spectrum of compound **2e**

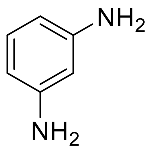

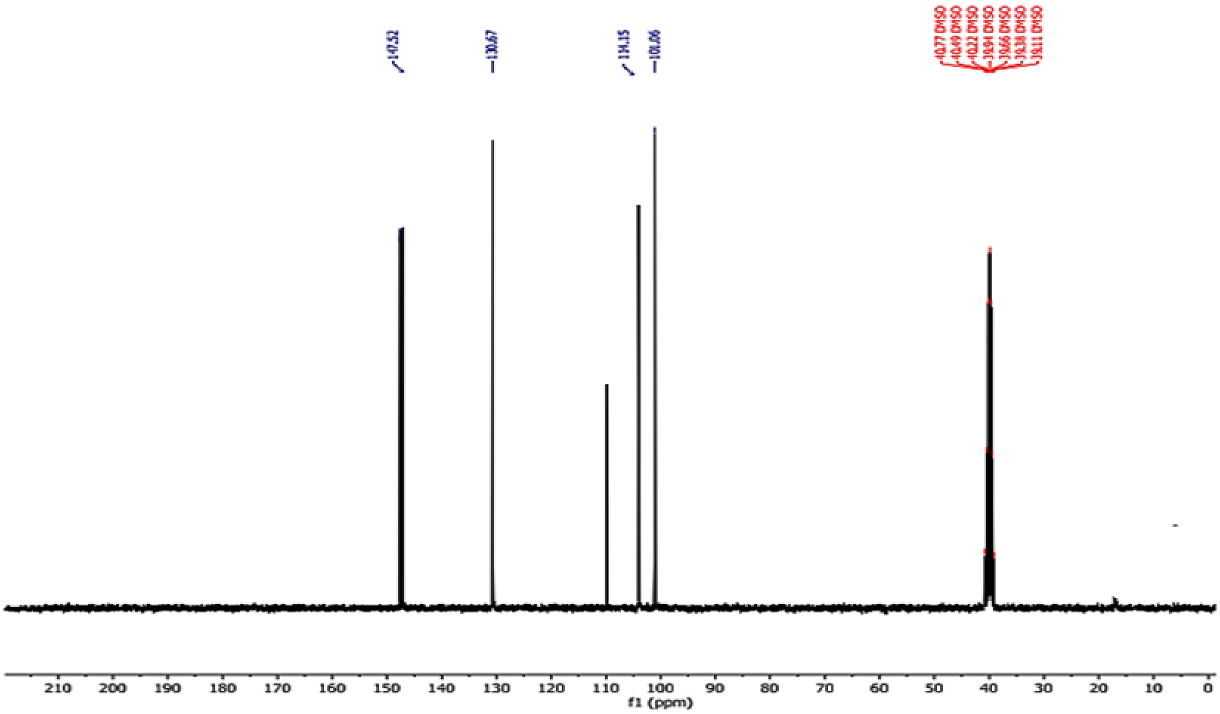

^{13}C NMR spectrum of compound **2e**

Benzene-1,4-diamine (2f)

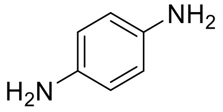

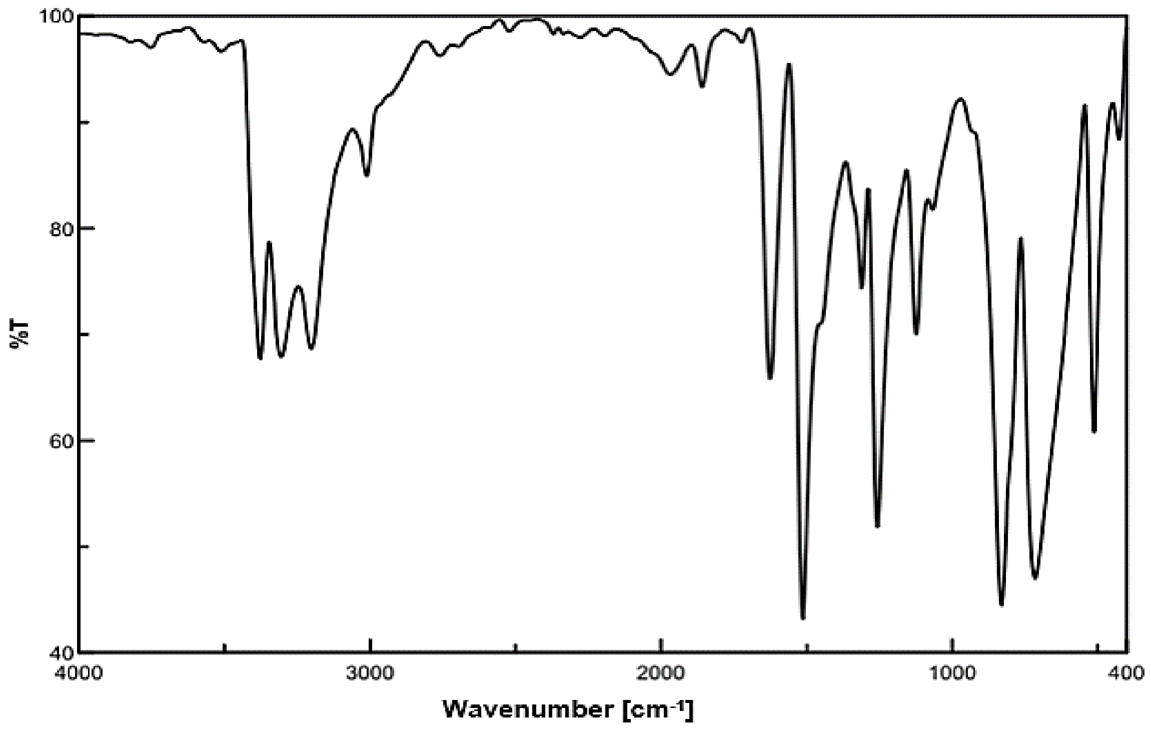

FT-IR spectrum of compound **2f**
